# Neonatologists’ Resuscitation Decisions at Birth for Extremely Premature Infants. A Belgian Qualitative Study

**DOI:** 10.3389/fped.2022.852073

**Published:** 2022-03-24

**Authors:** Alice Cavolo, Bernadette Dierckx de Casterlé, Gunnar Naulaers, Chris Gastmans

**Affiliations:** ^1^Department of Public Health and Primary Care, Centre for Biomedical Ethics and Law, KU Leuven, Leuven, Belgium; ^2^Department of Public Health and Primary Care, Academic Centre for Nursing and Midwifery, KU Leuven, Leuven, Belgium; ^3^Pregnancy, Fetus and Newborn, Department of Development and Regeneration, UZ Leuven, Leuven, Belgium

**Keywords:** ethics, neonatology, extremely premature infants, neonatal intensive care, qualitative study

## Abstract

**Objective:**

Deciding whether initiating or withholding resuscitation at birth for extremely preterm infants (EPIs) can be difficult due to uncertainty on outcomes. Clinical uncertainty generates ethical uncertainty. Thus, physicians’ attitudes and perspectives on resuscitation of EPIs might influence resuscitation decisions. We aimed at understanding how neonatologists make clinical-ethical decisions for EPI resuscitation and how they perceive these decisions.

**Methods:**

We performed a qualitative study using a constructivist account of grounded theory. Face-to-face, semi-structured in-depth interviews with neonatologists comprised data collection. For data analysis, we used the Qualitative Analysis Guide of Leuven.

**Results:**

We interviewed 20 neonatologists working in 10 hospitals in Belgium. Participants’ decision-making can be described as consensus-based, gestational age-based, contextualized, progressive, and shared. All participants agreed on the importance of using the consensus expressed in guidelines as a guidance for the decision-making, i.e., consensus-based. Consequently, all 20 participants use GA thresholds indicated in the guidelines, i.e., GA-based. However, they use these thresholds differently in their decisions. Few participants rigidly follow established thresholds. The vast majority reported using additional contextual factors as birthweight or parents’ wishes in the decision-making, rather than only the EPIs’ GA, i.e., contextualized. All participants agreed on the importance of involving the parents in the decision-making, i.e., shared, and indeed parents’ wishes were among the most valued factors considered in the decision-making. However, the extent to which parents were involved in the decision-making depended on the infant’s GA. Participants described a gray zone in which parents’ were viewed as the main decision-makers due to the high clinical uncertainty. This mean that participants tend to follow parents’ request even when they disagree with it. Outside the gray zone, physicians were viewed as the main decision-makers. This mean that, although parents’ wishes were still considered, counseling was more directive and the final decision was made by the physician.

**Conclusion:**

Although an EPI’s GA remains the main factor guiding neonatologists’ resuscitation decisions, other factors are seriously considered in the decision-making process. All neonatologist participants agreed on the importance of involving parents in the decision-making. However, they involve parents differently depending on the EPI’s GA.

## Introduction

Extremely premature infants (EPIs) are infants born earlier than 28 weeks of gestation (1). Although many treatment decisions are necessary during the whole care trajectory for these infants, the first decision to be made is, generally, whether to initiate resuscitation at birth (2). A systematic review of outcomes reported the following survival rates (mean 95% CI) for EPIs born in high-income countries. Survival rates are reported by gestational age (GA), i.e., the number of complete weeks of gestation counting from the last menstruation. The survival rates are: 7.3% of all live births at GA 22 weeks; 25.7% at 23 weeks, 53.9% at 24 weeks, 74% at 25 weeks, around 80% at 26 weeks, and 90% at 27 weeks (3). These infants also have a higher risk of mild (e.g., behavioral disorders) to severe (e.g., blindness, cognitive impairments) disabilities compared to infants born at term (4–7). The risk of severe disability decreases as GA increases (3). However, other factors influence the individual chances of survival with good quality of life (3, 5, 7, 8). These factors can be fetal (e.g., fetal growth, congenital anomalies, gender), clinical (e.g., administration of corticosteroids) or contextual (e.g., pharmaceutical and technological equipment available at the hospital, local resuscitation guidelines) factors (3, 5, 7, 8).

Resuscitation at birth of EPIs is an ethically sensitive decision. Because of this complex interplay of factors, determining the specific chances of survival with good outcomes of each individual infant can be difficult (9, 10). Such clinical uncertainty inevitably raises ethical questions. For example, is life expectancy always to be increased as much as possible or is it in the best interest of the baby to withhold treatment and ensure a short but painless life? Who should make the decision? The combination of clinical and ethical uncertainty can make it difficult understanding when resuscitation is appropriate. Consequently, physicians’ attitudes toward resuscitation of EPIs can also influence resuscitation decisions (9–11). Therefore, understanding neonatologists’ perspectives about the decision-making for resuscitation of EPIs can improve our understanding of such a complex decision-making.

Most studies on neonatologists’ perspectives of EPI resuscitation at birth have employed quantitative methods (11). While these provide a useful overview of what neonatologists do currently, or would do, they provide little insight into *how* neonatologists make decisions in practice and *how* they perceive those decisions (11). The few qualitative studies suggest that neonatologists find decisions about resuscitation of EP infants ethically difficult (12–15), especially because of the great clinical uncertainty (12) and because of parental involvement (13, 14, 16). Moreover, most of these studies focused primarily on the perspectives of parents and other healthcare providers, rather than solely on neonatologists’ perceptions (12, 14–16). A deep and nuanced understanding of neonatologists’ decision-making in such ethically-sensitive situations is still lacking. Thus, we conducted a study in Belgium aimed at understanding how neonatologists make clinical-ethical decisions regarding resuscitation of EPIs and how they perceive these decisions.

## Materials and Methods

### Study Design

We performed a qualitative study, employing a constructivist view of the Grounded Theory (17, 18). This design consists of using qualitative empirical data to generate theories that explain a phenomenon as opposed to use preconceived theoretical theories or frameworks. Research data are generated by the interaction between the participants, the researchers, and their environment (17). As such, it is particularly indicated for eliciting rich descriptions of complex phenomena as neonatologists’ clinical-ethical decision-making in actual cases. We followed the Consolidated Criteria for Reporting Qualitative Research guidelines (19).

### Setting

Belgium is a federal state consisting of three autonomous regions: Flanders, population, 6,629,143; Wallonia, population, 3,645,243; and Brussels, population, 1,218,255 (20). At the time of this study, a total of 113 neonatologists were practicing in 19 NICUs across the three regions. Flanders is the only region with an official guideline about when to resuscitate EPIs based on GA (21) advising resuscitation from 26 weeks and non-resuscitation below 24 weeks unless parents specifically request resuscitation after being well informed. Between 24 and 25 weeks, decision-making is done case-by-case through shared decision-making between parents and clinicians and considering all relevant factors, not GA alone. NICUs in Wallonia and Brussels have their own written or oral institutional recommendations. The majority of these institutional guidelines use the same GA thresholds of the Flemish guideline. Only two Walloon guidelines use a 24-week threshold: From 24 weeks resuscitation is mandatory; below 24 weeks resuscitation is generally not recommended but exceptions are considered.

### Participants and Recruitment

Participants were considered eligible if they (1) were neonatologists currently working in a NICU in Belgium, (2) were involved in resuscitation decisions involving EPIs, (3) were willing to participate, and (4) to be interviewed in English.

We used two recruitment strategies. First, the president the Belgian Society of Neonatology sent an invitation email and an information brochure (Supplementary Material 1) to all society members, i.e., gatekeeper method (22). Second, recruited participants invited colleagues who showed interest in participating, i.e., snowball method (22).

Interested neonatologists were asked to sign a written informed consent form and to complete a questionnaire. The questionnaire consisted of closed-ended questions on neonatologists’ socio-demographic characteristics, e.g., age, work experience, religion; on hospital’s characteristics, e.g., private or public, number of beds; and on previous experiences with this type of decision-making, e.g., whether they ever accepted a resuscitation/non-resuscitation request from parents. Following the General Data Protection Regulation and the KU Leuven regulations, we were only allowed to collect relevant data. Hence, we chose which characteristics to include in the questionnaire based on the results of a previous literature review (11). The questionnaire enabled us to ensure that interested neonatologists were eligible and to select participants with a variety of personal and hospital characteristics to ensure a diverse sample representative of real life.

After returning the questionnaire and the informed consent, neonatologists were contacted by the interviewer to give them the opportunity to ask for more information and to schedule an appointment.

### Data Collection

Data were collected by means of individual, face-to-face, semi-structured interviews byAC, a 30- year-old, PhD student with a background in philosophy and bioethics. Data collection started in September 2019 and ended in October 2020 when saturation was reached.

We developed the interview guide based on two previously published literature reviews (23, 24) and a pilot interview. As we wanted to understand neonatologists’ decision-making in real practice, we asked participants to describe past cases in which they had to decide whether to resuscitate EPIs at birth and we did an in-depth discussion of the decision-making for that family. We refined the interview guide further throughout the data collection process using preliminary results obtained from the interviews we conducted. The last version of the interview guide can be found in Supplementary Material 2.

Interviewees could choose the location of the interview. All interviews occurred at the participants’ hospital. Only the interviewer and participant were present in the room. If someone had to access the room, the interview was temporarily suspended. All interviews were conducted in English due to the interviewer’s lack of proficiency in Flemish and French. The interviews lasted, on average, 1 h with range: 37–82 min and were audiotaped after explicit consent from participants. No field notes were taken.

### Data Analysis

**FIGURE 1:**
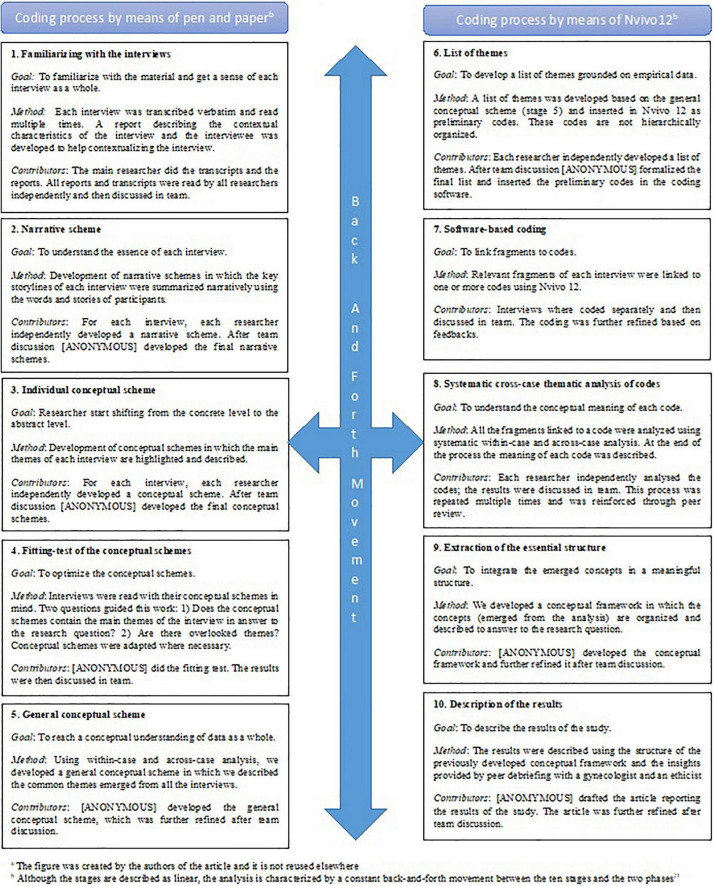
Analysis process by means of Qualitative Analysis Guide of Leuven (QUAGOL) (20, 21)*^a^*. *^a^*The figure was created by the authors of the article and it is not reused elsewhere. *^b^*Although, the stages are described as linear, the analysis is characterized by a constant back-and-forth movement between the 10 stages and the two phases (21).

Consistent with Grounded Theory, data collection and analysis occurred simultaneously. The Qualitative Analysis Guide of Leuven (QUAGOL) (25, 26) was used. This method is dived into two parts: Coding process by means of pen and paper, and coding process by means of software (we used Nvivo12). Each part is further subdivided into five steps. An interdisciplinary team composed of the interviewer, an ethicist, and an expert in qualitative research conducted the analysis. The different steps of the QUAGOL were first conducted independently by the three researchers and then discussed in team until consensus was reached. The QUAGOL is characterized by a combination of within-case and across-case analysis. This approach allowed us to identify the uniqueness of each case, while contextualizing it within the broader circumstances of the other cases. A more detailed explanation of each step of the analysis is provided in Figure 1.

To increase trustworthiness of the findings we used the systematic and interdisciplinary approach of QUAGOL; bracketing, which consists of acknowledging possible biases and addressing during the research; and place triangulation, i.e., we included 10 NICUs from the three Belgian regions to avoid overrepresentation of neonatologists from one region. Finally, we conducted peer debriefing: two external experts reviewed our data, analysis, and results, and gave us insights in what we might have overlooked or not explained well. This was performed by and ethicist and a gynecologist. We invited an ethicist with expertise in clinical ethics and pediatrics because of the clinical-ethical focus of the study. We invited a gynecologist because gynecologists are often the ones referring women at risk of premature pregnancy to a neonatologist.

### Ethics

The study received ethical approval from the ethics committee of the authors’ institution.

The informed consents procedure was rigidly followed. Participants received all the relevant information before participating in the study and were given multiple opportunities to ask additional questions regarding the study or the research group. All participants signed a written informed consent form prior to participation in the study. At the beginning of each interview, participants were asked to confirm their willingness to participate in the study and they were reminded that they have the right to withdraw at any point without need for explanation.

Confidentiality of personal information and anonymity was guaranteed in accordance with the General Data Protection Regulation of 25 May 2018. Participant identity was protected, and the data were anonymized.

## Results

### Demographics

Twenty-one neonatologists returned the questionnaire and informed consent form. We excluded one neonatologist based on the exclusion criteria. In total, we interviewed 20 eligible neonatologists. They were working in 10 NICUs spread across Flanders (*n* = 5), Wallonia (*n* = 3), and Brussels (*n* = 2). Participants’ age ranged from 34 to 63, and their years of experience in an NICU ranged from 2 to 30. Most of them were involved in resuscitation decisions for more than 5 extremely preterm births in the year prior to participation. Although qualitative research does not aim at find significant correlations, we considered whether personal or hospital characteristics seem to be more or less associated with response patterns. We did not find particular associations between characteristics and response patterns. However, the study sample was homogeneous in terms of ethnicity (20/20 white), gender (15/20 female), and religious affiliation (15/20 Roman Catholic). Summaries of the participants’ characteristics and their affiliated NICUs are presented in Table 1.

**TABLE 1:** Demographics of participants and characteristics of affiliated NICUs*^a^*.

Participant characteristics (*n* = 20)	No.
**Sex**	
Male	5
Female	15
**Age, y**	
30–35	2
36–40	4
41–45	2
46–50	5
51–55	1
56–60	4
61–65	2
**Religious affiliation**	
Roman Catholic	15
Liberal/no affiliation	5
**Years of experience in NICUs (excluding years of training)**	
1–5	3
6–10	4
11–15	2
16–20	3
21–25	4
26–30	3
>30	1
**Self-reported resuscitation cases in the previous year**	
0	1
1	1
2	4
3	3
4	2
≥ 5	9
**NICU characteristics (*n* = 10)**	**No.**
**Belgian region**	
Flanders	5
Wallonia	3
Brussels	2
**Hospital type**	
Academic	5
Non-academic	5
**Number of beds in unit**	
15–20	4
21–25	0
26–30	4
31–35	1
35–40	1

*^a^The table was created by the authors of the article and was not used elsewhere.*

### Neonatologists’ Clinical-Ethical Decision-Making

Our analysis of participants’ clinical-ethical decision-making revealed five dimensions describing the decision-making process: consensus-based, GA-based, contextualized, progressive, and shared. These dimensions can be considered as the pillars of the decision-making. However, they are not independent from each other; rather, they are intertwined and aspects of each often overlap (for quotes that exemplify these themes, see Table 2).

**TABLE 2:** Illustrative quotes: neonatologists’ clinical-ethical decision-making*^a^*.

Consensus-based	I think we tried to individualized medicine but individualized on the patient not on the doctor! I think we as doctors. I mean to me as chief of service is really important to build a team and to align the team on consensus. I think it’s very important because it’s never good to… We have big responsibilities in our hands and I think it’s important that we are aligned, we are aligned, that we all agree on what we do. At least on the what: on what we do, and the how can vary from person to person. I can use different words for parents, I can hear different things from parents as an individual, but I think we should at least have a framework of agreement on the direction and the parameters of what we do (Part.16) I think it was a huge progress to have a uniform policy and to be able to discuss cases in the team but also between units [.] It made the counseling much more clear for everyone because now it’s not depending on the person who’s giving the counseling to the parents what will be said and what will be the decision. And that’s because we are all following the same policy or consensus. (Part. 8)

GA-based	*Why is it so important to have a limit?*^b^** Ah it’s a big question. I think it’s important because for the parents it’s easier to say “after 24 weeks we will take care and before only if you ask us because it’s very difficult.” I think it’s easier inside a team, we should have a common point of view and for a common point of view you need a limit. (Part 12) *And did it ever happen that a parent asked for non-resuscitation above 26 weeks?*^a^** It happens, but that’s a no. There are too much data now that the chance to the good prognosis is high enough to try. (Part 2) *What are for you the main differences between inside the gray zone and outside the gray zone?* The main difference is the chance of a good life. In the gray zone we are less sure about the chances of good life for this child and his family. So that’s why we as doctors and as caregivers are not sure if we are doing something good by giving this child a chance. […] That’s the reason why it’s a gray zone so then you have to discuss it more and try to consider different aspects. (Part 17)

*GA-based/strict adherence*	He was a little boy, he had a very good weight, I think he was 600 g and he was breathing, no! I lied. He wasn’t breathing but he had a very good heart rate. But obviously we were not going to resuscitate that baby. We had already told the parents, we have already decided, it was a 22 weeks we were not doing anything. […] that was quite hard. But I mean, there was no way with a 22 weeker with an infection that we would have resuscitated that baby. That wasn’t really a resuscitation but we have a lot of things like that: 22 weeks that are little fetuses but that’s not for resuscitation (Part.19) (*Referring to the case of twins of 25 weeks in very good conditions but parents refused resuscitation*) we could not get to the point where the parents chose active treatment and there was the guideline! Then if there is a guideline that says “we take the decision together with the parents” you cannot say I discard the opinion of the parents eh?! […] that’s not possible, because of the guideline! In that case you can only wait and see and support the baby that they are comfortable and not in pain. (Part. 15)

*GA-based/flexible adherence*	*And is this an improvement for you? (having an open protocol)* Yes, of course. Because the very rigid protocol: do not even think at 24 weeks. It looks easier because there’s less questions but then other questions arrive what we do with maturation, transfer, palliative care if we don’t see the mother? And bla bla bla so there was a lot of unknown answers. So it’s much better but it’s more complex because it’s open. So when there’s a 24 weeks or 23 weeks it’s difficult because you don’t know what you have to do (Part.1) *If you are called to contribute to a guideline for resuscitation of prematures, what would be your advice?* I would advise against simple binary cut-offs, so should we put the threshold at 23 or 24? That’s not a good way to put it. I think things are more complex so it should be more articulated. So I would say gradual thresholds. For example steps, and conditions to each step. And I would really emphasize the reasonable factor. So the factor that the threshold is an indicator not a law. It’s not a biological law and in such a case the reasoning is a factor in itself (Part.16) *What’s the difference between a protocol and a guideline? (after the participant said that she liked the current guideline because it is not a protocol)* Well, a protocol is really “you have to do”; a guideline is “we suggest to do this, and this, and this.” I mean a protocol is really like “hemoglobin is 10 then you do this.” For these things you can’t have a protocol, you have to have a guidance “best is to do this, we suggest to do this.” (Part 17)

Contextualized	So I think that’s really for the gray zone, we factorize again development, is the development adequate for the age, malformation, is there any complication? Infection? Anything that you think may significantly worsen the prognosis that influences your decision. And the parental perception with the caveat that I really believe that parents should be involved in the decision, but it is challenging for the parents to actually make an informed decision because there are many barriers. […] so you know there’s a lot of variables. So especially in the gray zone we really have to do our best to understand all the variables and to really be able to share that with the parents. (Part 16) I think sometimes children who are 26 but with enterocolitis and growth restriction and only a weight of 400 or 500 g… Yeah sometime a 24 weekers with good weight are better off than the older ones who didn’t grow well. You have to see it in his context. It’s not really the number only but it’s everything around (Part. 11)

*EPI-related factors*	*First of all what are your conditions for non-resuscitation?* I cannot say them explicitly, but if you have a baby of 25 weeks with a lot of congenital malformation who will need 3/4 cardiac operations, then I would say “do you really want to do that? No” So it depends. If it is only prematurity and the baby has no other. then of course why not?! I think we should start. But if there are many extra things that make the situation worse… (Part. 11) For me personally, when it’s <450 g starts to be an ethical problem, and before 24. Then depending on the prenatal ultrasound, if there’s any malformation associated, and then based as I was saying on the extra-uterine adaptation: if the baby shows signs of vitality or not at all, and then of course also the parents’ will. (Part. 3)

*Parents-related factors*	It depends on the circumstances. In the second case, the parents were married for 15 years, they were trying to have a baby for 15 years. So it’s quite different from a lady of 18 years who discovered 2 weeks ago that she’s pregnant. I can understand that such a lady would not ask to do everything for the baby, and I can hear it. And for the other one I can hear that they want everything to be done. (Part 12) Then you realize that the most important in the follow-up is the family, is the social class, is the education level, is all the things that shouldn’t be. […] You can’t say “ok I won’t reanimate anyway because you are a poor family, and you can’t educate him. Forget about him.” I think you shouldn’t do this. You should try to help these mothers to cope with, to try to find money, resources, and so on. Not saying “ok you’re the bad statistics, we don’t…” (Part.1)

*(Lack of) time*	Sometime we have parents that say “a premature child is gonna be a handicapped child and I don’t want you to touch the baby before 28 weeks” and it’s really difficult. Then we try to talk and come to an agreement. All this when you have a mother who is in the maternal intensive care, and you have the time to discuss! In urgent situations we do our best and the parents have almost no part in the decision-making. (Part. 17) (*Comparing two cases: one in which she had time to counsel the parents and one in which she did not*) It’s easier for me because the mother has been hospitalized for 2 weeks before the birth, and we met the mother with the gynecologist. I think they received a good information: knowing the risk, knowing that it could be difficult for the baby and difficult for the parents. For the first little girl, I think everything was going so fast that we didn’t have the time to give a good explanation to the parents. And the fact that we had no time to have pulmonary maturation…. It’s the only intervention that shows that the survival rates are bigger after birth if we had time to give corticosteroids to the mother. (Part. 12)

Progressive	I had the possibility to stay in dialog, and to defend or to discuss my own act. I think it’s very important that you have something from prenatal, then the birth, then the postnatal, that makes the feeling that you can care for these parents and this child as a whole. […] Even if a child has to die it’s the same: that you can just take the discussion before the birth and also the aftercare. Also in the aftercare with a child that died is really important that you still have this whole thing with the parents. (Part. 17) When you speak of extremely premature babies there’s a big difference: you see parents, and usually you discuss what we’re gonna do. But the baby is not there. Sometime I say that to the mother “it’s like saying to someone who is gonna take his car what are we gonna do when you crash on the road. And then you have the team coming on road, and seeing someone who has a crash. This is very different.” So it’s very tricky to speak with someone about what you’re gonna do if you deliver today or in 1 week, if she’s 23 but maybe she’s 24, if she looks like 500 g but maybe she’s 600 g. So you have to be really cautious with the limits you’re going to give before the accident. (Part. 1)

Shared	The decision-making really needs to be shared with the nursing team and the parents because they are really the primary actors. There’s much more personalized involvement with the baby and with the parents and nurse so there are actually more protagonist in the story. (Part. 16) We talk a lot with each other, we have a good team! If I have something difficult ethically, I always talk with a couple of colleagues, then it is better. You cannot always stick on the things that go wrong, sometime you have to say “it’s like that, you cannot change it, you have to accept it.” It helps if you have a good team spirit. […] I think that’s important for a neonatologist. If you have a bad team, UH! That’s terrible I think because so much heavy things happen and if you have the feeling that you cannot talk with anyone because uh they are not nice. then that’s very difficult. But here with all my colleagues I have the feeling that if there is something I can talk about it and they do also talk with me. We help each other in the decisions. (Part. 11) There was also the presence of the obstetrician. I think it helps- it helped- quite a lot because finally they have more continuous contact with the parents. (Part. 3)

*^a^The table was created by the authors of the article and was not used elsewhere.*

*^b^We reported in italics the questions of the interviewer.*

#### Consensus-Based

All interviewed neonatologists said it is important to reach a consensus on resuscitation of EPIs. On a broader level, there should be a general consensus on when to resuscitate EPIs and on parental involvement within the unit and possibly within the whole community of neonatologists in Belgium. This type of consensus benefits the neonatal community as a whole because sharing the same points of reference facilitates discussion within and across units. According to the participants, consensus also benefits the child and the family because it contributes to improve the care provided. Consensus creates consistency of care between professionals avoiding the possibility that whether a child is resuscitated only depends on the personal views of the neonatologist present at birth. It also creates consistency of care across hospitals avoiding that resuscitation practices change drastically from one hospital to the other with the consequence that the survival rates would also drastically change depending on the hospital in which the child is born. Consequently, reaching consensus also improves the counseling given to parents. Specifically, it avoids the situation where parents might receive different information from different doctors, which can be confusing for parents during this delicate decision-making process. However, not everyone agreed that such consensus should be expressed in an official written guideline. Few participants preferred a consensus in the form of oral agreement because it would provide consistency while leaving room for individual evaluations.

On a case level, participants stressed the importance to reach a consensus for each individual premature birth. This consensus should be achieved among all the relevant stakeholders in the care of each individual infant. First and foremost, consensus should be achieved with the parents. All participants agreed on the importance of involving the parents in the decision-making to reach a decision that is shared and agreed upon. In fact, lack of consensus with the parents was perceived as a major ethical challenge. Second, consensus should also be achieved within the care team, including all the specialists following the family such as gynecologists, psychologists, and social workers.

Regarding case-level consensus, we noticed different attitudes depending on the units. In the majority of NICUs pre-delivery consensus was decisive. Consensus was reached pre-delivery with the team and the parents, and the neonatologist who was present during the delivery acted accordingly. This means that the attending neonatologist followed the consensus even if he/she personally disagreed with such decision. These participants explained that given the complexities of these cases, disagreements can occur. However, once the parents agreed on a course of action, it would be unfair to reopen the decision at birth, unless parents explicitly asked for it or the infant’s conditions were drastically different from what they expected. By contrast, in few NICUs pre-delivery consensus was indicative. The attending neonatologist used the pre-delivery consensus at birth to decide with the parents whether to resuscitate the infant. These neonatologists explained that you cannot obtain a complete information on the conditions of the infant before birth and, therefore, the final decision is made at birth by the attending neonatologist and the parents.

#### GA-Based

Consensus regarding resuscitation decisions at birth for EPIs is expressed practically in GA-based guidelines. For more information about Belgian guidelines, see *Setting*. All participants used GA thresholds indicated by the guidelines to some extent. The majority of participants (14/20) said that they generally agreed with the GA thresholds indicated by the guidelines because they align with clinical-statistical data. In their words, data show that from GA 26 weeks, chances of survival and good quality of life are sufficiently favorable to attempt resuscitation. Below GA 24 weeks, the chances of good outcomes are too low to justify resuscitation attempts. In-between these extremes, there is a “gray zone” in which outcomes are so uncertain that it is difficult to give general indications and, therefore, the majority of participants agreed that parents should be the main decision-makers. However, six participants would prefer compulsory resuscitation starting from GA 25 weeks instead of GA 26 weeks as currently indicated by the guidelines. In their opinion, international data show that outcomes are good enough to warrant an attempt of resuscitation.

Although participants generally agree with their guideline, they have different ways of using it in the individual cases. We identified a spectrum ranging from strict to flexible adherence to the guideline. At one end of the spectrum, five participants interpreted the guideline as a rigid protocol. Consequently, they rely strictly on the GA thresholds to make decisions. At the opposite end of the spectrum, four participants interpreted the guideline as a recommendation. For them the thresholds are always indicative rather than binding. The rest of participants situated themselves somewhere in the middle of this spectrum: They are flexible on the lower threshold, i.e., they all resuscitated infants born below GA 24 weeks, but they indicated an upper threshold, above which they would always resuscitate. It is important to note that these attitudes seemed to be at least partially influenced by the unit culture. In more flexible units, participants felt free to interpret the guidelines more flexibly and make exceptions, whereas in more rigid units they tend to make less exceptions. Five participants explicitly stated that they would prefer a much more flexible approach that would allow them to refuse a non-resuscitation request from parents at 25 weeks if the condition of the child is good but that they rigidly follow the guideline on this point because this is the consensus in the unit/region.

#### Contextualized

All participants considered contextual factors other than GA to some extent. They explained that GA is only one of the many contextual factors influencing an EPI’s prognosis and, consequently, their decisions for resuscitation. We identified three groups of factors that influence neonatologists’ decision-making: EPI-related factors, parent-related factors, and time or lack thereof.

EPI-related factors are all the clinical aspects, other than GA, that improve or worsen the child’s prognosis. Examples made by participants include birthweight, congenital anomalies, and whether antenatal steroids were administered. Generally, participants consider these factors along with GA and make counseling and recommendations based on the overall prognosis of the infant.

Parent-related factors are parents’ wishes and all those characteristics of the parents that may be relevant for decision-making such as the mother’s gynecological history, and previous experiences with miscarriages or stillbirths. We observed that parents’ experiences that generate empathy can greatly influence neonatologists’ decisions about resuscitation. This type of contextual knowledge is highly valued by many neonatologists, because it helps them to decide and to act in an empathic way. For example, one neonatologist explained that he decided to resuscitate an infant at 22 weeks despite knowing it would have been unsuccessful –for the parents. During decision-making, the parents revealed that they had already had a child at 22 weeks who was not resuscitated. The neonatologist said that it was clear to him that they would have not been able to cope with a second loss under the same circumstances unless they knew that they had exhausted all possibilities to support a premature birth. Cases like this one suggest that sometimes parent-related factors can be more influential in the decision-making than the EPI-related factors.

By contrast, nine participants perceived parents’ socioeconomic status as an ethical challenge for decision-making. These participants were aware that parents’ socioeconomic status could potentially influence an EPI’s future outcome. However, they felt uncomfortable with using this fact, and they were uncertain to what extent they should consider socioeconomic status or even whether it is appropriate to consider it in resuscitation decisions.

The last influencing factor is time. It is important to premise that for participants the ideal ethical standard would be building a relationship with parents and involving them in the decision-making through comprehensive and individualized counseling. To do so they need enough time to counsel parents as many times as necessary, to let parents discuss with each other, and to listen to them. Another important aspect was to have the time to discuss not only the technical aspects of the decision but also to get to know parents, to understand their wishes, hopes, fears, values etc. However, in cases in which the mother arrived at the hospital already in labor, either because the parents were not referred to the neonatologist in time or because it was an emergency delivery without previous symptoms, this was difficult to achieve. Participants explained that, although they tried to involve parents as much as possible, they had to make decisions with limited counseling and limited parental involvement. Although these situations were rare, they were perceived as highly challenging. This is why they perceive lack of time as a hindrance to an informed decision-making, which, in turn, was often perceived as a hindrance to an ethical decision-making. Because of that, many stressed the importance of starting counseling in a timely fashion.

The importance given to contextual factors suggests that participants make resuscitation decisions based on a much more complex interplay of factors rather than on GA alone. Participants used contextual factors in different ways in their decision-making. Those who strictly adhere to the consensus tend to consider other contextual factors only in the gray zone, when the guidelines suggest deciding whether to resuscitate on a case-to-case basis. Those who have a more flexible adherence to the consensus tend to always take contextual factors into account. For example, they would consider non-resuscitation above GA 25 weeks if the infant’s growth were restricted, if the infant has congenital problems, and if the parents ask for non-resuscitation. Again, the majority of participants sit somewhere in between. These doctors tended to consider contextual factors not only in the gray zone, but exceptionally also at GA 22–23 weeks. By contrast, contextual factors are almost never considered above GA 25 weeks.

To conclude this section, we want to mention two external factors: unit culture and neonatologist-related factors. These factors are not related to a specific case and they are not actively considered by the interviewees in the decision-making, rather they might be influenced by them. Regarding unit culture, participants did not explicitly mention it. However, we noticed that participants working in NICUs with a more flexible approach to the guideline tended to have a more flexible approach as well. Participants working in NICUs with a stricter approach tended to apply the guideline more strictly. Few participants also said that they would adopt a more flexible approach if it was not for the current guideline. This suggests that there might be a relation between unit culture and decision-making. Regarding neonatologist-related factors, none of the characteristics included in the demographic questionnaire seemed to be related to a specific response.

#### Progressive

Resuscitation decisions are perceived as part of a decision-making process that develops over time rather than a single one-moment decision. This process ideally starts before the EPI’s birth with counseling, it continues throughout the whole care trajectory, and it is constantly reassessed based on contextual factors. Indeed, all participants agreed that they should revise the decision when necessary, e.g., when the pregnancy progresses, when new data become available, or when parents change their minds. Even after resuscitation, the decision is still seen as revisable in the sense that it is possible to withdraw treatment if the child’s condition worsen. The fact that resuscitation is revisable whereas non-resuscitation is definitive lead five participants to prefer resuscitation when clinical uncertainty is high or when parents disagree with each other.

#### Shared

Participants concurred that resuscitation decisions are shared firstly with the parents, then with the team of neonatologists, and finally with other clinicians. All participants (20/20) agreed that parents should always be involved to some extent in the decision-making process regarding resuscitation of their children for the following reasons. First, treatment decisions for EPIs can affect the mother’s care, for example if a C-section for fetal indication is necessary. Participants said that treatment decisions might also influence parents’ long-term wellbeing. Second, the majority of participants (14/20) acknowledged parents’ right to make decisions for their children. Finally, participants acknowledged that the child’s interest is intertwined with the interests of the family. However, how parents are involved and the extent of such involvement depends on the guidelines and the GA of the infant. We will describe parental involvement more in depth in discussing the relation between clinical-ethical decision-making, perceptions of decision-making, and counseling.

Fifteen participants acknowledged the importance of the team of neonatologists in the decision-making. These participants stressed the importance of not making decisions alone. Ideally, they preferred pre-delivery team discussion, including neonatologists and other clinicians, followed by discussion with the parents. Such team discussions were valuable to understand the better course of action from a clinical and ethical point of view, especially for difficult cases. Even during night/weekend shifts, when there was less personnel available, or during emergencies, when there was less time available, they tried to gather at least a second professional opinion. In these cases, they either phoned the on-call colleague or with the other clinicians present at delivery. They also stressed the importance of the team emotional support in dealing with difficult situations. Team support was deemed crucial when they faced disagreements with the parents’ decision or when the child died.

Finally, participants valued inputs from other professionals, with the most cited being the treating gynecologist or obstetrician (14/20). The gynecologist can provide them with relevant knowledge on the mother’s gynecological history and on the family as a whole, including their values, previous experiences, etc. Moreover, often the gynecologist is the one referring the mother to the neonatologist and indeed lack of good communication and cooperation between the two specialties was perceived as a hindrance to the decision-making and to the maternal and child care in general. Other professionals cited to help with resuscitation decisions were psychologists and social workers, although they specified that once the child is admitted to intensive care the decision-making includes numerous other specialists. Overall, interdisciplinary team discussion was deemed extremely valuable both in preparation of delivery and after delivery during the NICU care trajectory.

### Clinical-Ethical Decision-Making and Counseling

We observed that the ways neonatologists make decisions are reflected in their counseling practices (see Table 3).

**TABLE 3:** Illustrative quotes: clinical-ethical decision-making and counseling*^a^*.

**Content**	*But why is it so important that there is consistency across*^b^**. Because those parents need to live with this decision. And I hope they got all the information that they needed, and they don’t find out that they didn’t receive some information that they were supposed to have. Or that they maybe perceive it differently because I said differently, or the receiver received it like they understood something that I didn’t meant. (**Part. 6**) And that is also something I learned to tell “don’t be afraid, maybe give it a chance, because at any moment if we see that there’s bleeding, or maybe things aren’t going well, and there’s a situation in which that doesn’t look good the future, we still can stop.” And when I tell that, a lot of parents, you can see that they are thinking again. That it’s not like” now we are going for it and there’s no way back,” they need to know that also. (**Part. 11**) One thing that is very difficult is the moment you go to see the couple, and you know that how you use your words will influence as well. For example in that second case, when I saw the couple the first time I was very negative, and I had the feeling I had to be very careful because I was influencing. Especially the one who was more in favor of not doing everything. I think the more difficult part ethically is to present all the medical information in an objective way. This is quite touchy almost in every case. (**Part. 3**)

**Way how**	I think the parents have to be talked to, have to be communicated to. We have to listen to their needs, their wants, their desires, their fears. It’s very very important. And again you have this range of parents. Some who don’t understand, or some that aren’t very educated, and some who understand very well. But their role is still very important. And their wishes! Like for example, the 50 years old mother even though the outcome for that 22 weeks baby was not- it wasn’t even an option- it’s still important to listen to her, to listen to her role, to listen to her story. Very important. (**Part. 19**) They (*the parents*) are not in the most serene position to make decision. They’re scared, there can be pain, labor, I mean many thing that prevent you to really be yourself. And then there are other factors very important, like in this institution 30% of our patients are non-Belgian. A lot are from either the middle east or north Africa. So they have different cultural background, and religious background, and language! So how do you talk with this people? Or how much do they get of what you say rather? I mean if you talk the same, are they as well informed as a Belgian patient? Certainly not! So you know there’s a lot of variables. (**Part. 16**)

*^a^The table was created by the authors of the article and was not used elsewhere.*

*^b^We reported in italics the questions of the interviewer.*

Participants described counseling as the tool to involve parents in the decision-making. All participant concurred that information delivered to parents needs to be complete, clear, and objective. Five participants admitted that, although they always strive for objectivity, if they believed the child would not survive, they had a more negative attitude, which could influence parents. As a manifestation of consensus-based reasoning, eight participants emphasized that physicians in a team should present information consistently, otherwise it can be confusing for parents. Contextualized reasoning was also manifested in counseling practices, as nine participants explained that information needed to be individualized to address the specific needs of parents. For example, five participants explained that when parents are extremely worried about negative outcomes, they emphasize that they will withdraw treatments, should it be ineffective.

Contextualized clinical-ethical decision-making was also apparent in the way information was delivered to parents. The majority of participants agreed that it was important to be attentive to parents’ needs and to meet them through counseling, for example by providing an interpreter. Eleven participants also discussed the importance of understanding when parents are confused, scared, overwhelmed; and addressing these feelings. Five participants also explained that it was important to be honest with parents, including admitting clinical uncertainty.

Finally, we observed that counseling was also progressive. All participants agreed that counseling should start before birth and continue throughout the whole decision-making process. Some participants admitted that this way of counseling takes time and poses some practical challenges, but they viewed it as a truly ethical way of counseling parents. Six participants said that they prefer to counsel parents with the gynecologist because he/she knows better the parents.

### Clinical-Ethical Decision-Making, Perceptions of Decision-Making, and Counseling

Clinical-ethical decision-making, perceptions of decision-making, and counseling practices appeared to be related. Guidelines and GA of the EPI greatly influenced whether the decision was perceived predominantly as medical or parental (Table 4).

**TABLE 4:** Illustrative quotes: Clinical-ethical decision-making, perceptions of decision-making and counseling*^a^*.

**Parental decision**	I mean in that gray zone, for me, the counseling, because it’s yes or no, it can be closed. So you explain them what you know, the risks, the possible outcomes, the worries… then you leave the decision to them because it’s their decision. It’s informed decision and it’s shared because you are helping them to make the decision but you are not really making a decision in their place and sometime you need a second talk but normally after 1 or 2 talks the decision is made and you can close it. You just go for one or the other. Even if it’s the gray zone, once the decision is made is not gray anymore. (**Part. 8**) It is a gray zone so we as doctors are not sure we are doing a good thing by keeping such a child alive so then I think the emotional impact, the social aspect come into the balance more important than in a zone where you as a doctor know you are doing something right. I think that in the balance the emotional impact and the psychosocial comes higher in the gray zone. (**Part 17**)

**Medical decision**	A lot of different things enter into consideration but we talk to them (*the parents*) before to be able to feel what would be the right option for the parents and then we go and “these are the options.” Sometime they are very clear options and sometime not. And then with all the medical discussion and the parental discussion we try to find the best option: do we resuscitate? do we not resuscitate? Maybe one time we’ll arrive that every 23 weekers we will resuscitate, who knows, but we aren’t there yet. (**Part. 4**) She was at 28 weeks so that was a complete different situation (*compared to the previous baby who was 25 weeks and not resuscitated*) and I explained to her the risks for 28 weeks but there was no decision-making on whether or not to start intensive care we start anyway at 28 weeks. (**Part. 2**)

*^a^The table was created by the authors of the article and was not used elsewhere.*

The majority of participants (15/20) perceived resuscitation decisions in the gray zone to be a parental decision. Many participants described the decision-making in the gray zone as shared between the neonatologists, the parents, and, when time allows it, other specialists. However, the majority explained that with shared they really meant that clinicians give parents information and parents make the final decision. More specifically, participants said they needed to support parents and to help them make a decision “they can live with.” Consequently, in the gray zone parent-related contextual factors, especially parents’ wishes, were the most weighted factors. Counseling was viewed as a tool to aid parents’ make such a decision. Participants felt that they had to respect an informed parental decision, even when they disagreed with it. Respecting the consensus, respecting parents’ autonomy and acknowledging that parents had to live with the consequences underpinned this respect.

In EPIs older than GA 25 weeks, resuscitation was perceived as a medical decision by all participants. They would always resuscitate given the high chance of survival and good quality of life. Here, EPI-related factors were the most influential factors for all participants. In fact, even the four participants open to exceptionally not resuscitating above 25 weeks, would do so mainly based on EPI-related factors. Because it was a medical decision, counseling was more directive, mainly informing parents of the situation. However, they still aimed to foster a relationship with the parents, understand their feelings, and tried to help them cope.

In EPIs younger than GA 24 weeks, the final decision was also perceived as a medical one, and counseling tended to be more directive, with majority of participants advising non-resuscitation. However, 15 participants were open to make exceptions if parents explicitly asked for resuscitation. In this case, participants would consider parents’ wishes along with other contextual factors, including their opinion on which factors were more relevant in that specific case. Put simply, these participants would not consider resuscitation below 24 weeks if parents did not ask for it, but the fact that parents asked for it did not mean that they would automatically resuscitate the child. For example, one participant received a resuscitation request for twins at 22 weeks GA. She agreed to resuscitate the first twin, because its clinical condition was positive, and it was very important for the parents to try. However, she refused to resuscitate the second twin, because he was thought to have little chance of survival. In this regard, this is still a medical decision because even though they are open to make exceptions if parents wish so, the final decision is of the neonatologist. Below the gray zone, whether EPI-related factors are weighted more than parent-related factors depends on the characteristics of the case and on the attitudes of the individual neonatologist.

## Discussion

Our study had three main results. First, our interviewees stated that they follow the regional or unit guidelines when deciding whether to attempt resuscitation, which are all GA-based. This is consistent with numerous empirical studies showing a correlation between infants’ GA and physicians’ willingness to resuscitate (23). The majority of participants generally do not resuscitate EPIs earlier than GA 24 weeks, they do resuscitate later than 25 weeks, and they let parents mainly make the decision at GA 24–25 weeks. Worldwide, the majority of guidelines recommend active treatment from GA 25 weeks onward and place the gray zone between GA 23–24 weeks (27). This implies that Belgian neonatologists use a higher threshold compared to international standards. Although the majority of participants tend to conform to the guidelines, some expressed discomfort in accepting non-resuscitation requests at 25 weeks. These participants wish that the Belgian guidelines would soon change to make resuscitation attempts mandatory from GA 25 weeks conforming to the international guidelines and data.

The debate on resuscitation at birth of EPIs does not only question at which GA we should resuscitate, but also whether GA in itself is an appropriate criterion to guide resuscitation decisions (24). In fact, some authors argue that GA alone is not sufficient to determine EPIs’ real chances of survival and, therefore, relying solely on GA to make resuscitation decisions means failing to treat some potentially viable infants (28–30). These authors advocate for a decisional model based on all the relevant prognostic factors rather than GA alone (28, 31–34). This is partially in agreement with our findings. Most of our participants were aware that GA is not the only relevant factor. In fact, despite the Belgian guidelines generally advise non-resuscitation at 23 weeks, the almost totality of participants were open to resuscitate infants born at this GA based on relevant factors including, but not limited to, other clinical factors, and parents’ wishes and history. As participants in this study pointed out though, this prognosis-based approach promoted by the above mentioned authors, is not always feasible due to time constraints. In fact, it relies on extensive counseling with parents, which is not always possible if parents are not referred to the neonatal team in time or in those rare occasions in which the delivery is sudden and unexpected. This approach requires good collaboration between neonatologists, gynecologists, and obstetricians to guarantee timely referral and timely counseling. Moreover, although very rare, sudden deliveries not accompanied by previous symptoms or risks associated with preterm birth do exist and were perceived as extremely challenging by participants. Therefore, it is important to develop a strategy to deal with these situations appropriately.

A second finding of our study relates to parent-related factors. Parents’ wishes and parents’ characteristics that generate empathy were considered particularly important. By contrast, many participants found parents’ socioeconomic status a difficult factor to consider. Despite being aware of the influence of this factor on EPIs’ future development (35–37), they reported not knowing whether it is ethical to consider it in the decision-making. Some participants were even uncomfortable talking about it. Other studies on the impact pf parents’ characteristics on decision-making for resuscitation of EPIs focused mainly on the impact of race, marital status, or financial resources and as such were not directly comparable with our results (38–40). However, another Belgian study on nurses’ and neonatologists’ perceptions on end-of-life decisions found that some struggle to consider parents’ socioeconomic status and that others reflect on its ethical implications (41). Therefore, further research is needed to better understand problems and implications related to the influence of parents’ socioeconomic status, and how to support physicians in dealing with it during decision-making.

Finally, all participants stressed the importance of involving parents in the decision-making through extensive, sensitive, and individualized counseling that addresses parents’ specific needs. This is once again in line with the literature. Many studies have shown that although all parents want to participate in decision-making for the care of their children, they have different preferences regarding the modality and the extent of their contribution (14, 42–44). However, we noticed that the way in which parents are involved in the decision-making strongly depends on the GA of the child. For EPIs outside the gray zone, interviewed neonatologists perceived the final decision as a medical one, meaning that parental counseling is more directive. Gray-zone decisions are mainly parental to the extent that many participants felt they should not interfere, even when they disagree. Interestingly, many participants described this decision-making as shared even though this way of counseling contrasts with the shared decision-making model advocated for in the ethical literature (34, 45–50). In the shared decision-making model, parents’ and physicians’ decisional power is equally balanced, whereas our results suggest that the weight of decision power bends toward parents or physicians depending on GA. One possible explanation that could explain the differences between the theoretical definition of shared decision-making and how it is applied in clinical practice is the existence of practical difficulties in employing appropriate shared decision-making (51, 52). A systematic review of facilitators and barriers in pediatric shared decision-making found that factors as lack of time, parents’ defensive or anxious attitudes, or healthcare providers’ lack of skills in shared decision-making can hinder a successful shared decision-making (52). These are all element mentioned also by participants in our study. Another explanation could be the fact that physicians have a different perception of shared decision-making than parents or ethicists (14, 16, 53). For example, many participants believed that shared decision-making consists of providing non-directive counseling and let the parents decide because this should be a parental decision. Hence, more research is warranted to understand neonatologists’ and parents’ perspectives and needs regarding shared decision-making, as well as possible barriers for implementing it in real-world practice.

### Strengths and Limitations

Our results are based on a relatively large, diverse sample of neonatologists, with 18% of all neonatologists practicing in three regions of Belgium with varied experience and ages, and a diversity of NICUs, with 10 out of 19 NICUs in (non-)academic hospitals with different bed capacities. Participants’ years of experience ranged from 2 to 38 years, and their age ranged from 35 to 64 years.

However, our sample was homogenous when it comes to ethnicity, gender, and religious affiliation. This might have limited the generalizability of the results. Moreover, we acknowledge a possible selection bias, since only neonatologists who were interested in the topic self-selected. Finally, the interviewer and the participants are not native-English speakers, which might have negatively impacted the interviews. The use of a different language might have hindered participants’ descriptions of complex experiences, thoughts, and emotions.

## Conclusion

Neonatologists’ clinical-ethical decision-making is firstly based on EPIs’ GA. However, when time allows for it, neonatologists agreed on the importance of parental involvement, the degree of which depends on the EPIs’ GA. Also counseling practices range from non-directive to directive based on the EPIs’ GA. Additional studies might directly compare the resuscitation attitudes of neonatologists and parents in Belgium with those in neighboring European countries sharing similar sociodemographic or having very different sociodemographic. The aim of such studies would be to better understand decision-making in the gray zone.

## Data Availability Statement

The datasets presented in this article are not readily available because the datasets generated and/or analyzed during the current study to protect the privacy of participants as well as other persons involved in the discussed cases. Requests to access the datasets should be directed to corresponding author.

## Ethics Statement

The studies involving human participants were reviewed and approved by UZ/KU Leuven (S62867). The patients/participants provided their written informed consent to participate in this study.

## Author Contributions

AC contributed to the study design, data collection, data analysis, drafted the initial manuscript, and reviewed and revised the manuscript. BD and CG contributed to the study design, data analysis, reviewed the manuscript, and provided mentorship. GN contributed to the study design, critically reviewed the manuscript for important clinical intellectual content, and provided mentorship. All authors approved and are accountable for the final manuscript as submitted.

## Conflict of Interest

The authors declare that the research was conducted in the absence of any commercial or financial relationships that could be construed as a potential conflict of interest.

## Publisher’s Note

All claims expressed in this article are solely those of the authors and do not necessarily represent those of their affiliated organizations, or those of the publisher, the editors and the reviewers. Any product that may be evaluated in this article, or claim that may be made by its manufacturer, is not guaranteed or endorsed by the publisher.

## Abbreviations

EPI: Extremely premature infants
GA: gestational age
NICU: neonatal intensive care unit
QUAGOL: qualitative analysis guide of Leuven.
