# Supplementary material for: Neonatologists’ Resuscitation Decisions at Birth for Extremely Premature Infants. A Belgian Qualitative Study
Source: Front Pediatr. 2022 Mar 24;10:852073. doi: 10.3389/fped.2022.852073 (PMC8989134; doi:10.3389/fped.2022.852073)
Supplement: Supplementary file 1 [file Data_Sheet_1.PDF]

Centre for Biomedical Ethics and Law

KU LEUVEN

Information brochure

For participants

What is neonatologists' ethical reasoning in the decision making for resuscitation at start of treatment for Extremely Premature Infants?

A qualitative study

Project

We would like to invite you to participate in a scientific study of KULeuven. We are looking for neonatologists with experience in the management of (non)resuscitation decisions at start of treatment for Extremely Premature Infants (EPIs).

The objective of this study is to understand neonatologists' ethical reasoning in the decision making at start of treatment for Extremely Premature Infants (EPIs), through their own practices, experiences, and opinions. This will result in a better understanding of such an ethically sensitive decision making and it may inform future guidelines on the management of EPIs.

In this brochure you will find the objectives of this study and the reasons why your participation is so important.

We thank you for your time. Your participation is precious. If you have any additional question do not hesitate to contact us.

Contact information:

Alice Cavolo
Main researcher

Centre for Biomedical Ethics and Law

Faculty of Medicine – KULeuven

Kapucijnenvoer 35 Box 7001

3000 Leuven

+32 16 19 33 73
Alice.cavolo@kuleuven.be

KU LEUVEN

Who will conduct the research?

This research is conducted at the Centre for Biomedical Ethics and Law of KULeuven. My name is Alice Cavolo. Since the 1st October 2018 I am conducting a PhD research on the ethical aspects of (non)resuscitation decisions at start of treatment for EPIs. My research is funded by FWO Flanders. My research is supervised by professor Chris Gastmans and co-supervised by professor Bernadette Dierckx de Casterlé and professor Gunnar Naulaers.

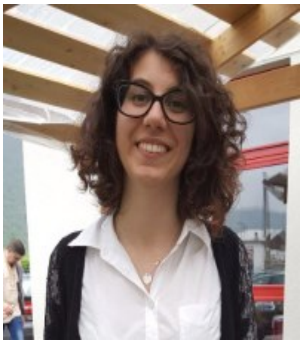

Alice Cavolo

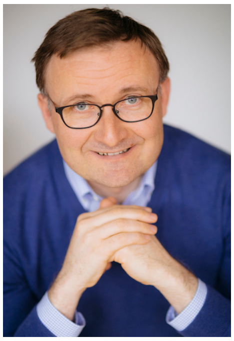

Prof. dr. Chris Gastmans

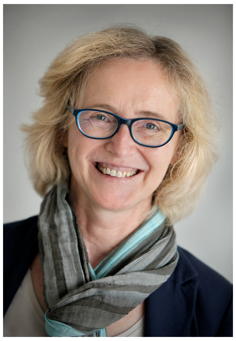

Prof. dr. Bernadette Dierckx de Casterlé

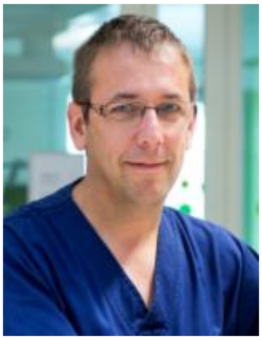

Prof. dr. Gunnar Naulaers

Background and Method

Extremely Premature Infants (EPIs) are babies born before the 28th complete week of gestation.

For them survival rates increase with the increase of gestational age. Other factors further influence mortality and morbidity of EPIs, such as gender, birth weight, twins, the use of prenatal steroids, the condition of the EPI at birth (e.g. the presence of congenital abnormalities), and the occurrence of postnatal events (e.g. sepsis, and intraventricular bleedings) making the individual prognosis difficult to assess and uncertain.
EPIs also have an increased risk of moderate or severe disability, which can include a wide range of physical and intellectual impairments, such as neurosensory (blindness or deafness), motor, cognitive, and behavioral impairments.

Deciding whether to resuscitate EPIs at start of treatment can be very difficult due to time pressure and uncertainty on the individual outcomes. Such uncertainty, in turn, raises ethical challenges such as:
Is life expectancy always to be increased as much as possible or is it in the best interest of the baby to withhold treatment and ensure a short but painless life?
Who should be involved in the decision making?
What should be done in case of disagreement between parents and physicians?

Given that, more insights in the dynamics and contents of the ethical decision-making is necessary.

Method

The scope of this qualitative study is to investigate neonatologists' ethical reasoning in the decision making regarding resuscitation at start of treatment of EPIs. Participants will be asked questions on their practices, experiences, and opinions.

We want to achieve this through in-depth individual face-to-face interviews.

Execution of the research and what is expected from you?

If you are willing to participate, we ask you to fill in and sign the attached informed consent form and questionnaire. You can return the documents by email. I will contact you to organize the interview.

The interview will last approximately 40-60 minutes and it will be carried out in English. If you agree, the interview will be recorded. The audiotape will only be used for the data collection and it will be destroyed at the end of the study.

KU LEUVEN

Rights of participants

Voluntary participation

You are totally free to choose whether to participate or not. You have the right to withdraw your participation at any time. During the interview, you can ask to suspend or interrupt the conversation in any moment. You do not have to give reasons for this decision and this cannot cause you any harm. You are not obliged to answer to all the questions.

Confidentiality

All personal information are strictly confidential. All information will be used exclusively for this study and will be carefully managed. Personal information will not be shared outside the research team.

Every participant will be assigned a code. With the exception of the main researcher who will conduct the interviews, the other members of the research team will only see the anonymized interviews. Moreover, Prof. Naulaers will not be allowed to see the interviews to ensure the privacy of his colleagues. Quotations used in publications will only be reported using the code and no personal information or information that may identify the participants will be reported.

We will not perform data collection per institute and to ensure privacy of participants their unit or any information that may identify their unit will not be reported.

Legal and ethical requirements

Confidentiality of personal information and anonymity will be guaranteed in accordance with the General Data Protection Regulation of 25 May 2018

This study has been approved by the Ethische Commissie Onderzoek UZ/KU Leuven. The committee has to protect the participants' rights and to ensure that all the legal and ethical requirements have been accomplished.

Costs and Compensations

The researchers will come to your working institution or at your place. Therefore, participation will not imply any cost and no compensation is given. However, if you have to travel to participate in the study, then the travel costs will be reimbursed.

Insurance

KULeuven has an insurance that guarantees that any damage resulting from your participation in this study will be reimbursed.

How can you participate in this study?

If you wish to participate, please:

- ✓ Read the information brochure
- ✓ Sign the informed consent form and complete the questionnaire
- ✓ Return the informed consent form and the questionnaire as soon as possible. You can send it:
  - via email at: alice.cavolo@kuleuven.be
  - Via postage at:

Alice Cavolo

Centre for Biomedical Ethics and Law
Faculty of Medicine – KULeuven

Kapucijnenvoer 35 Box 7001

3000 Leuven

- ✓ We will contact you as soon as possible to decide the place and time of the interview based on your own preferences.

We kindly thank you for your participation!

KU LEUVEN
