# Supplementary material for: Neonatologists’ Resuscitation Decisions at Birth for Extremely Premature Infants. A Belgian Qualitative Study
Source: Front Pediatr. 2022 Mar 24;10:852073. doi: 10.3389/fped.2022.852073 (PMC8989134; doi:10.3389/fped.2022.852073)
Supplement: Supplementary file 2 [file Data_Sheet_2.PDF]

INTERVIEW GUIDE

1. Presentation
2. Reference to the information brochure
3. Reference to the informed consent
4. Ask if they have further questions
5. Ask permission to record the interview

Introduction

Extremely Premature Infants (EPIs) are babies born before the 28th complete week of gestation. They generally need resuscitation which is a difficult decision. I am interested in the ethical complexities of deciding whether to resuscitate EPIs.

I firstly did a search of the empirical and ethical literature and I have obtained a good ethical and theoretical description of this decision-making. But I still don’t know how resuscitation decisions are made in practice from the perspective of the physicians who make this decision.

To do so I need to understand your experience, the challenges you met, how you dealt with them, and the reasons that motivate your choices. To understand that I need real cases.

I want to make to clear though first that I will remove any identifiable detail of the cases from the transcript. Second, that I will not judge the cases or your actions. I need these cases to understand the ethical challenges and how you deal with them in real practice not to evaluate id the decision was good or bad.

PART 1: CLINICAL CASE

WARMING-UP QUESTIONS

Look at the questionnaire and see if you want to ask something more there

- Do you have a hospital policy or guideline?
- Do you agree with it? What's your opinion on it?

- Why did you set the threshold for non-resuscitation at that week?

POSSIBLE OPENING QUESTION

(I will use one of the following opening questions depending on the results of the questionnaire)

Do you have a case above 25 weeks?

Do you have a case below 24 weeks?

Do you have a case that you perceived easy?

- Why is this case easy?
- What would be a difficult case?
- What are the factors that make a case easy or difficult?
- What if parents asked the opposite?

Based on the description of the case I will make questions to further understand the case and obtain the most comprehensive description possible.

Examples of such questions are:

Questions related to the clinical case

- What was your role in the decision making?
- What do you think of the situation?
- What was the main problem?
- What was the decision made?
- What were the alternatives?

Questions related to decision-makers reactions

- Can you tell me how did you feel in the decision making?
    - What factors complicated the decision making?
    - What factors facilitate it?
- Can you tell me how did the other caregivers react to the decision?
- Can you tell me how did the parents react to the decision?

Once we have a complete overview of the case I will make questions to achieve a deeper understanding of the following themes within the case. For each themes examples of questions are provided.

Ethical values

- Did you perceive any ethical issue?
- Which was the most important?

- How did you manage them?
- Do you think that resource allocation is a relevant factor to consider in this case?

- Do you think it was the right choice? / would you make the same decision? / are there things you would do differently?

Role of parents

- What was the role of parents?
- Do you think parents should have been more/less involved?
- Were you comfortable with parents' requests?

POSSIBLE QUESTIONS ON OTHER CASES

We will ask for other cases for a comparison. The specific question will change depending on the previous case description (for example if the first case was about an EPI outside the grey zone I will ask for a case about an EPI inside the grey zone)

Can you describe a case involving an EPI born inside/outside the grey zone?

Can you describe a case in which the decision making was more/less difficult?

- How does your decision-making change inside and outside the grey zone?
- What are the main differences?

- What makes this case more/less difficult than the previous one?
- Do you perceive the same ethical issues compared with the previous one?

- Why is your position different?

PART 2: Conclusions

POSSIBLE CONCLUDING QUESTIONS

If you were invited by the ethics committee of your hospital to participate in the development of ethics policy on (non)resuscitation of EPIs, what would be the key-message that should be integrated in the policy?

If you have to advise a young colleague on managing (non)resuscitation decisions/ non resuscitation requests, what would you advise?
